# Hepatic lead and copper concentrations in dogs with chronic hepatitis and their relationship with hematology, serum biochemistry, and histopathology

**DOI:** 10.1111/jvim.16149

**Published:** 2021-05-22

**Authors:** Eleonora Gori, Alessio Pierini, Valentina Meucci, Francesca Abramo, Luisa V. Muscatello, Veronica Marchetti

**Affiliations:** ^1^ Veterinary Teaching Hospital “Mario Modenato,” Department of Veterinary Sciences University of Pisa Pisa Italy; ^2^ Department of Veterinary Medical Sciences University of Bologna Bologna Italy

**Keywords:** biochemistry, canine, heavy metals, hematology, histopathology, liver

## Abstract

**Background:**

Although the influence of copper ([Cu]) on chronic hepatitis (CH) has been widely studied in dogs, little information is available about the accumulation of other metals.

**Hypothesis/Objectives:**

We assessed the concentration of lead ([Pb]) in the livers of dogs with CH with or without abnormal hepatic [Cu] to establish if any association existed between [Pb] and either hematologic or biochemical variables, fibrosis, necrosis and inflammation of the liver on histology.

**Animals:**

Thirty‐four dogs with CH that had hepatic [Cu] and [Pb] determined.

**Methods:**

Retrospective review of medical records of dogs with CH and hepatic [Cu] and [Pb]. Chronic hepatitis was defined using current American College of Veterinary Internal Medicine consensus statement guidelines. Hepatic [Cu] and [Pb] were determined using square wave anodic stripping voltammetry. Dogs were divided into 2 groups based on [Cu]: <400 ppm (LoCu) and ≥400 ppm (HiCu).

**Results:**

The median [Cu] and [Pb] were 357 ppm (range, 100‐7743 ppm) and 58.7 (range, 6.89‐224.4 ppm), respectively. Nineteen dogs had LoCu and 15 dogs had HiCu. Median [Pb] was significantly higher in HiCu compared to LoCu dogs (*P* < .001). Hepatic [Pb] and [Cu] were significantly correlated (rho = 0.7; *P* < .001). Dogs with microcytosis had higher [Pb] than did dogs with normal red cell volume (*P* = .02). Hepatic [Pb] was not correlated with either necroinflammatory or fibrosis scores.

**Conclusions and Clinical Importance:**

Although additional studies are needed to better understand the clinical role of hepatic [Pb], dogs with abnormal hepatic [Cu] may also have higher hepatic [Pb]. In addition, in dogs with high hepatic [Pb], microcytosis may be present.

**Abbreviations**
[Cu]: copper concentration
[Pb]: lead concentration
ALB: albumin
ALP: alkaline phosphatase
ALT: alanine transaminase
AST: aspartate transaminase
BIL: total bilirubin
CH: chronic hepatitis
CRP: C‐reactive protein
Cu: copper
GGT: gamma‐glutamyltranspeptidase
HCT: hematocrit
HGB: hemoglobin
MCV: mean corpuscular volume
Pb: lead
RBC: red blood cells
RDW: red blood cells distribution width
SWASV: square wave anodic stripping voltammetry
WBC: white blood cells
WSAVA: World Small Animal Veterinary Association

## 1 INTRODUCTION

In the last 15 years, the influence of copper (Cu) on liver disease, especially chronic hepatitis (CH), has been widely studied in dogs,^1^, ^2^, ^3^, ^4^, ^5^, ^6^, ^7^, ^8^, ^9^ but few clinical studies of other metal accumulations are currently available.^9^, ^10^ Both Cu and lead (Pb) may cause hepatic injury, because of defective hepatic metabolism or may be linked to oxidative stress mechanisms associated with their presence within hepatocytes, especially Pb.^3^, ^8^, ^9^ At toxic concentrations, free intracellular Cu initiates oxidative damage causing hepatocellular necrosis and inflammation.^3^, ^11^ Hepatic Cu accumulation can be associated with substantial hepatic injury resulting in acute hepatitis, CH, and cirrhosis.^11^, ^12^ A study on acute and chronic primary hepatitis found that copper‐associated hepatitis accounted for one‐third of all dogs with liver disease and approximately 35% of CH.^13^ The severity of hepatic injury also is correlated with hepatic Cu concentration ([Cu]).^3^ Hepatic [Cu] in normal dogs is between 150 and 400 μg/g dry weight (parts per million; ppm).^3^, ^14^ However, there is physiological variability in relation to breed and diet.^14^ The potential mechanisms for hepatic Cu accumulation include primary metabolic defects in hepatic Cu metabolism, cholestasis causing impaired biliary excretion of Cu, and excess daily Cu intake.^3^, ^15^

Data regarding hepatic Pb concentrations ([Pb]) in dogs are scarce.^9^, ^10^, ^16^, ^17^, ^18^, ^19^ In dogs, hepatic [Pb] mainly has been investigated as a sentinel for human exposure to heavy metals,^10^, ^16^, ^19^ but hepatic [Pb] recently was taken into account in dogs with hepatocellular carcinoma.^9^

We hypothesized that, in addition to [Cu], hepatic [Pb] also may play a role in hepatocellular injury and may be responsible for hematologic or histological alterations or both. Our aims were to: (a) evaluate hepatic [Pb] in dogs with CH with or without abnormal hepatic [Cu] and (b) establish if any association existed between [Pb] and either hematologic or liver histopathologic variables.

## 2 MATERIALS AND METHODS

Retrospective review of medical records at the University of Pisa Veterinary Teaching Hospital was conducted to identify dogs with CH, in which hepatic [Cu] and [Pb] also were measured. First, medical records were searched for dogs that had hepatic [Cu] and [Pb] measured. In our facility, dogs undergoing surgical hepatic biopsies undergo hepatic [Cu] assessment as part of routine evaluation for suspected CH. On the same sample, our toxicology laboratory measured [Pb]. The histological reports and formalin‐fixed paraffin‐embedded histology samples of these dogs then were reviewed by a pathologist with expertise in hepatic biopsy evaluation (Francesca Abramo) and a European College of Veterinary Pathology diplomate (Luisa Muscatello) and CH was defined using the current American College of Veterinary Internal Medicine consensus^14^ guidelines (Table S1) on slides stained with hematoxylin and eosin for histology and picrosirius red for fibrosis assessment. The histological features of CH include the presence of lymphocytic, plasmacytic, neutrophilic, and eosinophilic or granulomatous inflammation, along with hepatocyte necrosis and variable severity of fibrosis, lobular architecture distortion and regeneration.^14^, ^20^ Histological grading (necroinflammatory activity grading) and staging (fibrosis staging) of CH were applied to these histological samples.^20^, ^21^ All samples were scored for necroinflammatory activity and fibrosis according to the World Small Animal Veterinary Association (WSAVA) guidelines^20^ using the following scoring scheme, with necroinflammatory activity graded as A0 = absent, A1 = slight, A2 = mild, A3 = moderate, A4 = marked, or A5 = very marked.^18^, ^19^ Then, dogs were divided into necroinflammatory activity groups: A0‐1 (absent to slight) and A > 1 (mild to very marked).^20^ Again, using the WSAVA guidelines, fibrosis was graded as 0 = absent, 1 = mild, 2 = moderate, 3 = marked, or 4 = very marked, and based on fibrosis staging dogs were divided into F0‐2 and F ≥ 2.^20^, ^21^

Data on signalment, diet of the previous year and current diet, environmental data (urban, suburban or rural), and hematology and serum biochemistry variables were collected including red blood cell count (RBC), hemoglobin concentration (HGB), hematocrit (HCT), mean corpuscular volume (MCV), red blood cell distribution width (RDW), white blood cell count (WBC), reticulocyte count (Procyte DX, Idexx Laboratories, Westbrook, Maine), alkaline phosphatase (ALP), gamma‐glutamyltranspeptidase (GGT), alanine transaminase (ALT), and aspartate transaminase (AST) activity, and total bilirubin (BIL), albumin (ALB), and C‐reactive protein (CRP) concentrations (Liasys, Assel SRL, Rome, Italy).

Concentrations of Cu and Pb in liver biopsy samples were evaluated using an electroanalytical method based on square wave anodic stripping voltammetry (SWASV) coupled with an acid digestion protocol. Fifty milligrams of liver were treated with 2.5 mL H_2_O_2_ (33%); samples were heated to 50°C for 1 hour, cooled and then 2.5 mL of HCl (30%) was added. Samples then were heated to 70°C for 1 hour until a transparent solution was obtained. The volume of solution was brought to 5 mL with ultrapure water and purified by elution using Superclean ENVI‐Carb column to remove any organic residue. The Carb columns were preconditioned with 2 mL of methanol followed by 2 mL of ultrapure water, and the digested samples then were loaded and the column eluate collected in clean tubes. The limits of detection (LOD) of the studied metals were 0.5 and 0.3 μg/L for Cu and Pb, respectively. Considering a sample of 50 mg of liver, the detection limit was 10 mg/kg (ppm) for Cu and 6 mg/kg (ppm) for Pb.^22^ For calculations, a value corresponding to LOD/2 was assigned to all samples that had concentrations of hepatic [Pb] < LOD.^23^

### 2.1 Statistical analysis

Statistical analysis was conducted using IBM SPSS Statistics, v. 25 (IBM Corporation, New York, New York). All continuous variables were tested using the Kolmogorov‐Smirnov normality test, and non‐normally distributed variables were expressed as median, minimum‐maximum, and range, whereas normally distributed variables were expressed as mean ± SD.

For the main aims, dogs were divided into 2 groups based on hepatic [Cu]: LoCu = [Cu] < 400 ppm and HiCu = [Cu] ≥ 400 ppm^21^ and [Pb] was compared between LoCu and HiCu groups using an unpaired *t* test. The [Pb] also was correlated with [Cu] using Spearman's correlation test. Afterwards, [Pb] and [Cu] were correlated with all of the investigated hematologic and biochemical variables (RBC, HGB, HCT, MCV, RDW, WBC, ALP, GGT, ALT, AST, BIL, ALB, CRP) using Spearman's correlation test. Correlations were considered mild, moderate or strong with rho <0.3, between 0.3 and 0.6 or >0.6, respectively. In addition, [Pb] and [Cu] were associated with presence of anemia (RBC, HCT, or HGB or some combination of these below the reference range, 5.65 × 10^6^/μL, 37%, and 13.1 g/dL, respectively), microcytosis (MCV <61 fL), necroinflammatory activity score (A0‐1 and A > 1) and fibrosis score (F0‐1 and F ≥ 2) using a Mann‐Whitney *U* test. A *P*‐value <.05 was considered significant.

## 3 RESULTS

The initial population consisted of 38 dogs with CH. Three dogs were excluded because histology samples were not available for review, and 1 dog was excluded because blood test results were not available. The final cohort consisted of 34 dogs with CH, in which hepatic [Cu] and [Pb] were measured. Demographic information about the study population is presented in Table S2.

The mean age was 8.6 ± 3.4 years and there were 20 females (14 spayed) and 14 males (2 neutered). Most were mixed breeds (12 of 34; 35%), followed by Labrador Retriever and Cocker Spaniel (3 dogs of each breed), and Jack Russell Terrier (2 dogs). The remaining dogs belonged to the following breeds: American Staffordshire Terrier, Poodle, Standard Poodle, Dachshund, Beagle, Bolognese, Bernese Mountain dog, Boxer, English Bulldog, Dobermann Pinscher, Drathaar, Golden Retriever, German Shepherd, and Spitz (1 dog of each breed). All dogs lived in Tuscany and Liguria, the majority of which (21/34) lived in an urban environment, whereas the remaining 13 dogs came from suburban and rural environments (5 and 8 dogs, respectively). Twenty‐seven dogs of 34 (79.4%) were fed a branded commercial pet food (2 of which were adult maintenance diets, and the remaining 25 of which were prescription veterinary diets), whereas 3 dogs were fed a home‐cooked diet (chicken or turkey without offal), and the remaining 4 dogs were fed a mixture of commercial and home‐cooked diets without offal.

Necroinflammatory activity scores were assigned as follows: A0 = 1 dog; A1 = 10 dogs, A2 = 12 dogs, A3 = 5 dogs, A4 = 2 dogs and A5 = 4 dogs. Regarding fibrosis staging, 7 dogs had F0, 6 dogs had F1, 12 dogs had F2, 4 dogs had F3, and 5 dogs had F4.

**FIGURE 1:**
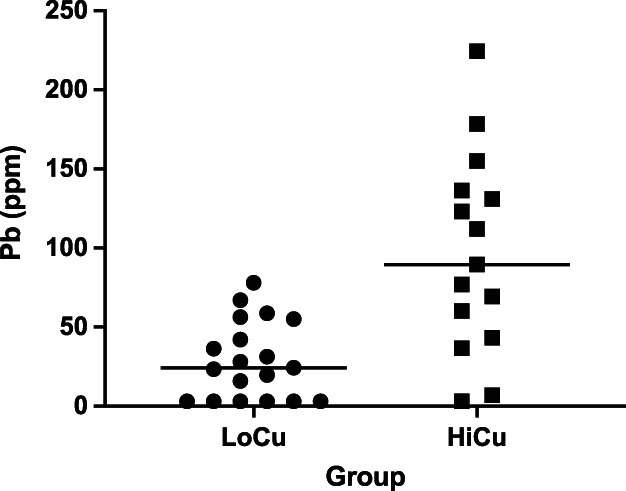
Scatterplots of hepatic [Pb] compared between dogs with hepatic [Cu] <400 (Group LoCu) and ≥400 ppm (Group HiCu; median 24.3 ppm vs 89.5 ppm; *P* < .001; Mann‐Whitney *U* test)

**FIGURE 2:**
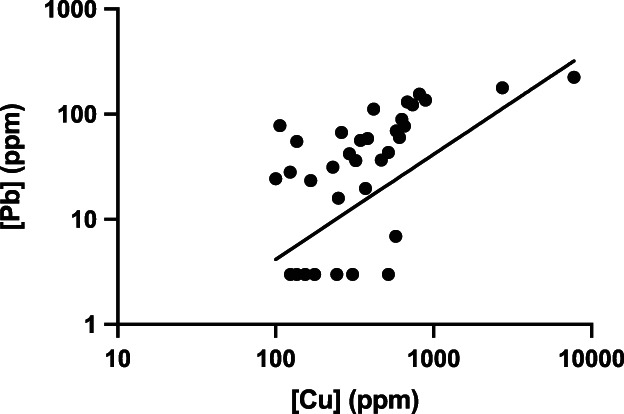
Correlation between hepatic [Pb] and [Cu] in dogs with CH (rho = 0.7; *P* < .001). Both hepatic [Pb] and [Cu] are expressed in log10 scale

Based on hepatic [Cu], 19 dogs (56%) belonged to LoCu (median [Cu] 230 ppm; range, 100‐381 ppm) and the remaining 15 dogs (44%) belonged to HiCu (median [Cu] 630 ppm; range, 417‐7743 ppm). Seven dogs had hepatic [Pb] below the LOD (6 dogs in LoCu and 1 dog in HiCu, respectively) and 3 ppm was assigned as [Pb], as described above.^23^ Median [Pb] was significantly higher in the HiCu compared to the LoCu group (89.5 ppm vs 24.3 ppm; *P* < .001; Figure 1). Hepatic [Cu] and [Pb] were significantly correlated (rho = 0.7; *P* < .001; Figure 2).

**FIGURE 3:**
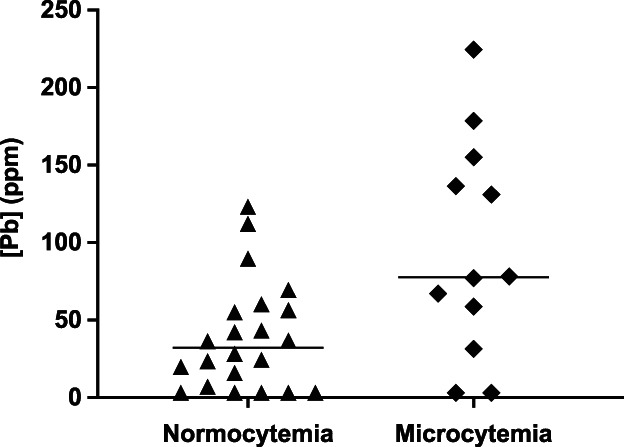
Scatterplots of hepatic [Pb] compared between normocytic dogs and dogs with microcytemia (MCV < 61 fL; median 77.5 ppm vs 32 ppm; *P* = .02; Mann‐Whitney *U* test)

Twelve dogs had anemia (35%), which was moderate in 1 dog and mild in the others. Twelve (35%) dogs had microcytosis. Neither hepatic [Cu] nor [Pb] differed between anemic and nonanemic dogs (*P* = .15 and *P* = .98, respectively). Dogs with microcytosis had higher median [Pb] (77.5 ppm) than did dogs with normal MCV (32 ppm; *P* = .02; Figure 3).

Correlations between hepatic metals and hematology and serum biochemistry variables are shown in Table S2. None of these variables correlated significantly with either [Pb] or [Cu]. Hepatic [Pb] and [Cu] were not different between necroinflammatory score groups (*P* = .99 and *P* = .42, respectively) and fibrosis grading score groups (*P* = .29 and *P* = .84, respectively).

## 4 DISCUSSION

We investigated hepatic [Cu] and [Pb] and their relationship with histopathological findings and hematology and serum biochemical variables in dogs with CH. We included dogs with CH so as to obtain the most homogeneous and standardized population^17^, ^18^ and to compare our data with previous studies on heavy metals in the canine liver. We found significantly higher hepatic [Pb] in dogs with hepatic [Cu] ≥ 400 ppm compared to dogs with [Cu] < 400 ppm. In addition, hepatic [Cu] and [Pb] correlated positively and hepatic [Pb] was negatively associated with MCV.

In our population, approximately the half the CH dogs had hepatic [Cu] > 400 ppm.

Copper‐associated hepatitis was first reported in Bedlington Terriers and it also has been described in West Highland White Terriers, Dalmatians, Doberman Pinschers, and Labrador Retrievers.^7^, ^24^ In these breeds, the hypothesized mechanism is a congenital defect, sometimes inherited, which alters Cu metabolism, resulting in abnormal accumulation of hepatic Cu (>1000‐1500 ppm) and secondary hepatitis.^7^, ^24^ Breed‐associated Cu accumulation may not be a reliable explanation for our results, because we only included 3 Labrador Retrievers, of the predisposed breeds, 1 of which had normal hepatic [Cu]. Moreover, the genetic defect in Cu metabolism has only been described briefly in these specific breeds.^7^, ^24^

Abnormal hepatic [Cu] also may occur as an acquired defect of excretion and metabolism because of the hepatic disorder itself. In fact, cholestasis, which may be a consequence of parenchymal hepatic disease, may lead to a decreased biliary Cu excretion and, consequently, its accumulation.^2^ This mechanism also has been proposed for hepatic accumulation of Pb, which has been studied in acute exposure in rodents^25^ and, in both acute as well as chronic environmental exposure, in small animals.^8^, ^14^, ^15^, ^16^, ^21^, ^22^, ^26^, ^27^, ^28^, ^29^, ^30^, ^31^, ^32^, ^33^, ^34^, ^35^, ^36^, ^37^, ^38^ In all of the scenarios described above, the simultaneous presence of Pb and Cu within the hepatocyte and the subsequent hepatic injury may trigger inflammation and oxidative stress.^2^, ^39^ In particular, oxidative stress is thought to be the main mechanism of hepatic injury caused by Pb.^25^, ^39^

Investigating hepatic [Pb] in relation to [Cu], median [Pb] was found to be significantly higher in dogs with [Cu] ≥ 400 ppm than in dogs with [Cu] below that threshold, and hepatic [Pb] and [Cu] were found to be strongly correlated. This is a novel finding because, to the best of our knowledge, no studies have investigated the relationship between hepatic [Cu] and [Pb]. To explain this result, we considered several hypotheses. Firstly, because the liver is the principal recipient of Cu absorbed from the gastrointestinal tract and is essential for its metabolism, the same may be true for Pb and Pb also may accumulate in the liver parenchyma. In addition, a proportional increase in the 2 metals may be explained as a detoxification defect as a result of hepatic injury and cholestasis or a combination of these factors.

Regarding Pb, in the last decade, domestic and wild animals have been studied as sentinels for toxicological risk assessment of humans.^15^, ^16^, ^26^, ^27^, ^28^, ^29^, ^30^, ^31^, ^32^, ^33^, ^34^, ^35^, ^36^, ^37^, ^38^ Companion animals especially have been studied as potential sentinels for environmental Pb exposure, because they share much of the same environment with people, and may even be more exposed than their owners to several contaminants (water, soil).^16^, ^21^, ^22^, ^26^, ^27^, ^28^, ^29^, ^30^, ^31^, ^32^, ^33^, ^34^, ^35^, ^36^, ^37^, ^38^ However, the majority of the these studies have been conducted on blood [Pb], in order to establish Pb exposure.^15^, ^21^, ^22^, ^27^, ^29^ The other studies, which performed [Pb] assessments in various organs, in particular the liver and kidneys, consisted of necropsy evaluations without any comparison with clinicopathological variables.^8^, ^14^, ^16^, ^28^, ^30^ The majority of veterinary clinical studies on Pb were performed in the 1970s and 1980s and focus on acute Pb intoxication in small animals, especially dogs.^34^, ^35^, ^36^, ^37^, ^38^ However, today, because of the European Commission's laws (EU Directive 2002/32/CE), Pb intoxication is rare in Europe. Furthermore, given that since the 1990s hepatic [Cu] has increased significantly over time in both purebred and nonpurebred dogs, the influence of diet on hepatic [Cu] cannot be ruled out.^40^ Two studies have already considered Pb traces in pet food.^41^, ^42^ In the first study,^41^ 12 different commercial veterinary diets were compared to supermarket brand pet food in terms of toxic trace metals. The results emphasized that the most undesirable metal elements in foods, such as Pb, were below detectable or quantifiable concentration.^41^ The second study, which was performed in the United States, analyzed various pet foods containing fish, red meat, or poultry.^42^ In terms of heavy metals, the Pb content in the analyzed pet foods was well below the toxic daily intake of Pb, suggesting a large margin of safety in pet foods.^42^ However, although our data included a small study population on which statistical analysis of this aspect was not performed, hepatic [Pb] still could play a role in liver disease.

Regarding the correlation between [Pb] and [Cu] and hematology and serum biochemistry variables, we found a significant association between [Pb] and the presence of microcytosis. This relationship has not been reported previously in dogs. In animals, hematologic abnormalities have been described in acute Pb toxicosis, the most common of which is anemia.^22^, ^25^ Other potential hematological findings include basophilic stippling, decreased myeloid‐erythroid ratio, and evidence of ineffective erythropoiesis in bone marrow.^22^ In contrast, in humans, chronic Pb intoxication is a cause of microcytosis, especially in children.^43^, ^44^ One of the main reasons why Pb toxicosis causes anemia and microcytosis is that Pb interferes with the activity of a specific enzyme, that is important in the biosynthesis of heme (delta‐aminolevulinic acid dehydratase),^45^ which may cause oxidative stress‐induced erythrocyte damage.^25^ However, the influence of chronic disease on RBCs and microcytosis cannot be ruled out.^46^

No significant association was found between hepatic [Pb] and any of the studied hematology and serum biochemical variables. This lack of significant findings could be explained by our study population. All of our dogs had CH, and thus all had various degrees of alteration in relation to their hematology and serum biochemistry variables. Because hepatic enzymes are dependent on the amount of liver parenchyma, the unknown influence of fibrosis and inflammation on residual liver parenchyma cannot be ascertained. Comparison could be made to a previous study^15^ on the Pb exposure of dogs living around a Pb mining area in Zambia, because of chronic exposure of this population to Pb.^15^ Surprisingly, and in accordance with our findings, in contrast to the high metal exposure found in the previous study, most of the serum biochemistry variable analyzed, such as ALP, AST, ALT, and GGT activity, and BIL, ALB, and total protein concentrations, were within or nearly within the standard reference interval.^15^ Such findings may indicate that chronic Pb exposure does not increase hepatic injury or alter hematology or serum biochemistry variables.

We failed to find a significant association between hepatic [Cu] and the necroinflammatory and fibrosis score. The literature contains contrasting information on hepatic [Cu] and histologic changes. As in our study, a previous study^47^ did not find a significant association between hepatic [Cu] and histological changes. In contrast, another study found that in dogs with CH, hepatic [Cu] was not significantly associated with the histopathologic fibrosis score but was associated with the inflammation activity score.^24^ Other studies aimed at correlating hepatic [Cu] with histology findings have taken into account a semiquantitative Cu accumulation based on rhodanine‐specific Cu staining.^2^, ^48^ The discrepancy between our data and the literature thus could be explained by the different method used (ie, atomic absorption spectrometry vs SWASV) and the population and period of time of case inclusion. In fact, the previous retrospective study^24^ covered a very wide time interval (1980‐2010), in which the histological classification used was a modified Ishak‐Knodell system developed by Cornell University, and their study population was entirely composed of Labrador Retrievers, unlike ours which was composed mainly of mixed breed dogs.

Our study had several limitations. First, the number of cases enrolled was small. In the future, the study population should be increased with a larger cohort of dogs with CH. Second, we may have inadvertently not assessed our histological findings correctly (inflammation and fibrosis). In fact, it has been found that pathological liver injury may include a nonhomogeneous distribution of lesions, and thus an underestimation of these variables cannot be ruled out.^19^, ^26^ Most importantly, in veterinary medicine there are no specific studies on the possible role of chronic Pb exposure on liver tissue or a specific threshold for hepatic [Pb], as is the case for [Cu], which could be used as a direct comparison for our data. In addition, because of the retrospective nature of our study, we were unable to evaluate blood [Pb], which may have added interesting information regarding hematological abnormalities.

## 5 CONCLUSION

Although additional studies are needed to better understand the clinical role of hepatic [Pb], dogs with abnormal hepatic [Cu] also may have higher [Pb] than dogs with normal hepatic [Cu]. This could be a result of concomitant accumulation, a direct consequence of hepatic Cu accumulation, or concomitant oxidative damage caused by increased [Pb]. In addition, in dogs with CH and high hepatic [Pb], microcytosis may be worse compared to dogs with CH and low hepatic [Pb].

## CONFLICT OF INTEREST DECLARATION

Authors declare no conflict of interest.

## OFF‐LABEL ANTIMICROBIAL DECLARATION

Authors declare no off‐label use of antimicrobials.

## INSTITUTIONAL ANIMAL CARE AND USE COMMITTEE (IACUC) OR OTHER APPROVAL DECLARATION

Authors declare no IACUC or other approval was needed.

## HUMAN ETHICS APPROVAL DECLARATION

Authors declare human ethics approval was not needed for this study.

## Supporting information


**Table S1** Necroinflammatory activity and fibrosis staging of chronic hepatitis according to World Small Animal Veterinary Association (WSAVA) guidelines (Van den Ingh et al. 2016).

**Table S2** Demographic characteristics of the study population of dogs with chronic hepatitis.

**Table S3** Correlations between hepatic metal concentrations and hematobiochemical parameters and necroinflammatory activity grade in 34 dogs with chronic hepatitis.
