# Supplementary material for: Hepatic lead and copper concentrations in dogs with chronic hepatitis and their relationship with hematology, serum biochemistry, and histopathology
Source: J Vet Intern Med. 2021 May 22;35(4):1773–9. doi: 10.1111/jvim.16149 (PMC8295653; doi:10.1111/jvim.16149)
Supplement: Supplementary file 1 — Table S1 Necroinflammatory activity and fibrosis staging of chronic hepatitis according to World Small Animal Veterinary Association (WSAVA) guidelines (Van den Ingh et al. 2016). [file JVIM-35-1773-s003.pdf]

Table S1. Necroinflammatory activity and fibrosis staging of chronic hepatitis according to World Small Animal Veterinary Association (WSAVA) guidelines (Van den Ingh et al. 2016).

Necroinflammatory Activity (Grade)			
**Grade**	Periportal or Periseptal Interface Hepatitis	Focal Lytic Necrosis	Confluent Necrosis
**Absent (0)**	Absent	Absent	Absent
**Slight (1)**	Very mild	1 focus per 10 × obj.	Absent
**Mild (2)**	Mild	2-4 focus per 10 × obj.	Absent
**Moderate (3)**	Moderate	5-10 focus per 10 × obj.	Absent
**Marked (4)**	Marked	>10 focus per 10 × obj. and/or→	Confluent or bridging necrosis
**Very marked (5)**	Marked	>10 focus per 10 × obj. and/or→	Bridging or panacinar/ multiacinar necrosis

A focus was defined as a discrete area of inflammation or necro- sis and interface hepatitis is defined as inflammation and erosion of the hepatic parenchyma at its junction with portal tracts or fibrous septa.

Degree of Fibrosis (Staging)			
**Stage**	Fibrosis	Bridging fibrosis	Bridging Fibrosis with Nodule Formation
**Absent (0)**	Absent	Absent	Absent
**Mild (1)**	Mild fibrous expansion (periportal or central)	Absent	Absent
**Moderate (2)**	Moderate fibrous expansion	Some bridging fibrosis (PP, CC, or PC)	Absent
**Marked (3)**	Marked fibrous expansion	Marked bridging fibrosis (PP, CC, or PC)	Absent
**Very marked (4)**	Marked fibrous expansion	Marked bridging fibrosis (PP, CC, or PC)	Present

PP, portal–portal; CC, central–central; PC, portal–central.
