# Supplementary material for: Hepatic lead and copper concentrations in dogs with chronic hepatitis and their relationship with hematology, serum biochemistry, and histopathology
Source: J Vet Intern Med. 2021 May 22;35(4):1773–9. doi: 10.1111/jvim.16149 (PMC8295653; doi:10.1111/jvim.16149)
Supplement: Supplementary file 2 — Table S2 Demographic characteristics of the study population of dogs with chronic hepatitis. [file JVIM-35-1773-s001.pdf]

Table S2. Demographic characteristics of the study population of dogs with chronic hepatitis.

Case number	Sex	Age (years)	Breed	[Cu] (ppm)	[Pb] (ppm)	Diet	F score	A score
**1**	MC	10	Cocker Spaniel	7743	224.4	Veterinary	4	5
**2**	MI	9	Golden Retriever	2730.4	178.4	Veterinary	4	1
**3**	FS	14	Standard Poodle	888	136.3	Mixed	1	2
**4**	MI	14	Bolognese	814	155	Veterinary	3	3
**5**	MI	9	Labrador Retriever	737.75	123	Veterinary	1	1
**6**	MI	13	Cocker Spaniel	682.14	130.9	Home-cooked	2	1
**7**	FI	8	Boxer	655.32	77	Home-cooked	1	1
**8**	MI	8	Poodle	630	89.5	Veterinary	0	2
**9**	MI	12	Labrador Retriever	609	60.11	Veterinary	1	2
**10**	FI	1	Jack Russell Terrier	582	69.4	Veterinary	2	1
**11**	FI	2	American Staffordshire Terrier	577	6.89	Maintenance	3	3
**12**	FS	9	Mix-breed	518.33	<6	Veterinary	0	1
**13**	MI	3	Mix-breed	517	43.2	Veterinary	0	1
**14**	FI	6	Mix-breed	467	36.7	Veterinary	4	5
**15**	FS	8	Mix-breed	417	112	Veterinary	4	5
**16**	FS	11	Dachshund	381.84	58.7	Home-cooked	1	4
**17**	MI	5	English Bulldog	370.12	19.7	Veterinary	0	0
**18**	MC	12	Mix-breed	345	56.32	Veterinary	2	2
**19**	FS	10	Mix-breed	322.27	36.3	Veterinary	2	3
**20**	FI	12	Mix-breed	306.74	<6	Mixed	2	2
**21**	FS	7	Mix-breed	293.5	42.1	Veterinary	2	1
**22**	MI	15	Mix-breed	261	67	Mixed	0	1
**23**	FS	11	Labrador Retriever	250	15.85	Veterinary	4	5
**24**	FS	9	Beagle	244	<6	Veterinary	2	3
**25**	FI	8	German Shepherd	230	31.3	Veterinary	3	2
**26**	MI	13	Spitz	177	<6	Veterinary	0	2
**27**	FS	7	Mix-breed	167	23.4	Veterinary	1	2

**28**	MI	8	Mix-breed	154	<6	Mixed	2	1
**29**	FS	7	Drathar	136	<6	Veterinary	3	4
**30**	FS	4	Cocker Spaniel	136	55	Veterinary	2	3
**31**	MI	7	Bernese Mountain Dog	124	<6	Veterinary	2	1
**32**	FS	7	Jack Russell Terrier	123.5	28.1	Veterinary	0	1
**33**	FS	5	Dobermann Pinscher	106.6	78	Maintenance	2	1
**34**	FS	9	Mix-breed	100	24.3	Veterinary	2	2

A score, necroinflammatory activity score (from 0 to 5); F score, fibrosis grading score (from 0 to 4); FI, female intact; FS, female spayed; MC, male castrated; MI, male intact; [Cu], hepatic copper concentration; [Pb], hepatic lead concentration.
