# Supplementary material for: Hepatic lead and copper concentrations in dogs with chronic hepatitis and their relationship with hematology, serum biochemistry, and histopathology
Source: J Vet Intern Med. 2021 May 22;35(4):1773–9. doi: 10.1111/jvim.16149 (PMC8295653; doi:10.1111/jvim.16149)
Supplement: Supplementary file 3 — Table S3 Correlations between hepatic metal concentrations and hematobiochemical parameters and necroinflammatory activity grade in 34 dogs with chronic hepatitis. [file JVIM-35-1773-s002.pdf]

Table S3. Correlations between hepatic metal concentrations and hematobiochemical parameters and necroinflammatory activity grade in 34 dogs with chronic hepatitis.

		[Pb] (ppm)	[Cu] (ppm)
RBC (M/µL)	rho	0.008	-0.25
	p-value	0.99	0.14
HGB (g/dL)	rho	-0.33	-0.28
	p-value	0.86	0.13
Hct (%)	rho	-0.12	-0.32
	p-value	0.48	0.07
MCV (fL)	rho	-0.28	-0.12
	p-value	0.09	0.5
RDW (%)	rho	0.30	0.13
	p-value	0.08	0.46
RETIC (K/µL)	rho	0.16	0.41
	p-value	0.42	0.06
WBC (K/µL)	rho	-0.23	0.08
	p-value	0.17	0.66
ALP (U/L)	rho	-0.22	-0.24
	p-value	0.21	0.18
GGT (U/L)	rho	-0.13	-0.05
	p-value	0.46	0.79
AST (U/L)	rho	-0.09	-0.23
	p-value	0.68	0.32
ALT (U/L)	rho	-0.09	-0.005
	p-value	0.88	0.98
BIL (mg/dL)	rho	-0.04	0.05
	p-value	0.80	0.79
ALB (g/dL)	rho	-0.02	-0.13
	p-value	0.88	0.49
CRP (mg/dL)	rho	0.23	0.13
	p-value	0.83	0.63
Necroinflammatory activity grade	rho	-0.07	-0.33
	p-value	0.69	0.85

Statistically significant correlations are reported in bold text. Abbreviations: [Cu], Copper concentration; [Pb], Lead concentration; ALB, albumin; ALP, alkaline phosphatase; ALT, alanine transaminase; AST, aspartate transaminase; BIL, total bilirubin; CRP, C-reactive protein; GGT, gamma-glutamyltranspeptidase; HCT, Hematocrit; HGB, Hemoglobin; MCV, Mean corpuscular

volume; RBC, Red blood cells; RDW, Red blood cells distribution width; WBC, White blood cells.
